# An Ergogenic Medical Education: Building Curricula to Optimize Performance and Decrease Burnout

**DOI:** 10.7759/cureus.17855

**Published:** 2021-09-09

**Authors:** Joshua Moen

**Affiliations:** 1 College of Health Education, Touro University California, Vallejo, USA

**Keywords:** performance enhancement, medical residency, professional burnout, medical education curriculum, health leadership and policy, physician leader

## Abstract

One of the most pervasive myths in our culture today is the belief that training increases performance. When, in fact, training decreases performance. The current structure of training programs and educational curriculums provide the evidence regarding the acceptance of this belief. Intense focus is placed on the quantity of training time with little regard for additional factors. In pursuit of excellence, maximizing training opportunity and learning exposure insists upon the sacrifice of recovery time. However, recovery is the necessary training period to increase performance. In athletics, training without recovery leads to overtraining syndrome. Burnout is the non-athletic equivalency seen in under-recovered learners and workers. As demonstrated by the climbing burnout rates, the current structure of educational programs, epitomized by medical residency, perpetuates the myth that more training equals better performance. The purpose of the article does not revolve around the presentation of novel research discoveries, but it insists upon the implementation of previously established performance data in curricula development beyond athletics. The inflection and deflection points along the growth and adaptation curves can be explicitly utilized to meet the educational and professional standards set forth by educational institutions. When tracking performance as the metric, initial training stimuli creates a descending slope, e.g., “training decreases performance.” The concept that training creates a negative deflection is a neglected concept in academics. By incorporating this feature into learning environments, training can transition from surviving training redundancy to thriving with an optimal work:recovery ratio.

## Introduction

Introducing the performance fallacy

To become the best in a given domain, an enormous amount of work must be put in. This requires dedication, commitment, sacrifice, and many hours of practicing. The climb to the elite level has become a one-size-fits-all race to the popular, but misinterpreted, belief known as the 10,000-hour rule. However, the firehose, quantity-based values of training is limiting performance.

The purpose of this article is to provide a more prosperous framework for developing the future leaders of medicine by utilizing established principles from sports science. Admittedly, on the first read, the difference may be considered a semantic argument. However, understanding the distinct temporal periods of growth and adaptation are critical to optimizing performance in trainees. To start, when discussing something like a medical residency training program, the benefits and harms are discussed from multiple perspectives. Matthew Alvin, MD., (2017) discusses The Individualized Comparative Effectiveness of Models Optimizing Patient Safety and Resident Education (iCOMPARE) trial from the perspective of fatigue [1]. Dr. Alvin deliberates the implications of 80-hour work weeks on residents, family, children, and even household pets. Others discuss the training implications and patient exposure opportunity regarding shift length and hospital time [2].

No matter the perspective, a common assumption within medical education and training is fatigue, burnout, and diminished free time are the necessary prices to pay to develop an expert leader in medicine. The following discourse will promote the imperative to rid our education systems, notably medicine, of the misguided myth of maximizing training hours to enhance performance. In order to cultivate the view fully, the ethical analysis of resident sleep deprivation and patient safety will be set aside. So too will the emotional and mental health impact of 80-hour workweeks. Both aspects of medical resident training have been thoroughly researched, discussed, and interpreted [3-6]. Instead, dismissing this myth involves expounding the ergogenic principles that underlie the mission of all medical residency programs.

## Materials and methods

Weight room gains: unexciting but necessary physiology

To understand the translation of sports science into medical curricula, an analogy will be utilized. Perhaps the most analogous concept outside of medical training is within the weight room. In this case, envision the objective of increasing the size and strength of the photogenic group of muscles that make up the quadriceps. One of the most frequented paths to accomplish this is through the use of back squats or leg extensions [7,8]; both of which, significantly activate the quadriceps and increase the cross-sectional area of the muscles [8,9].

While many factors known (and unknown) lead to muscle hypertrophy, a dominating theory is through a signaling pathway beginning at the z-disk. Serving as a mechano-receptor, any mechanical load on a muscle will be transmitted to the z-disk which is followed by activation of the anabolic pathway mTOR, satellite cell activation, and eventually an increase in protein synthesis rate, ribosome, and myonuclei quantity [10]. The minutiae of the hypertrophy response are unimportant to the scope of the paper, but the concept of muscle hypertrophy serves as a perfect example of training optimization. Wackerhage et al., (2019) describe the hypertrophy signal as an “initiating stimulus that is of a sufficient magnitude and duration to trigger a skeletal muscle hypertrophic response to resistance exercise” [10]. In other words, the aforementioned repetitions of leg extensions and squats were mechanistic signals for the future cascade of events leading to hypertrophy.

The importance of this concept cannot be overstated when translating its findings to ergogenics in education. After providing the physiological instructions through repetitions of leg press and leg extension, the process of growth is no longer under conscious control. The work has been done, the signal sent, and now in order for this signal to transmit and propagate, resources and recovery are needed.

**Figure 1:**
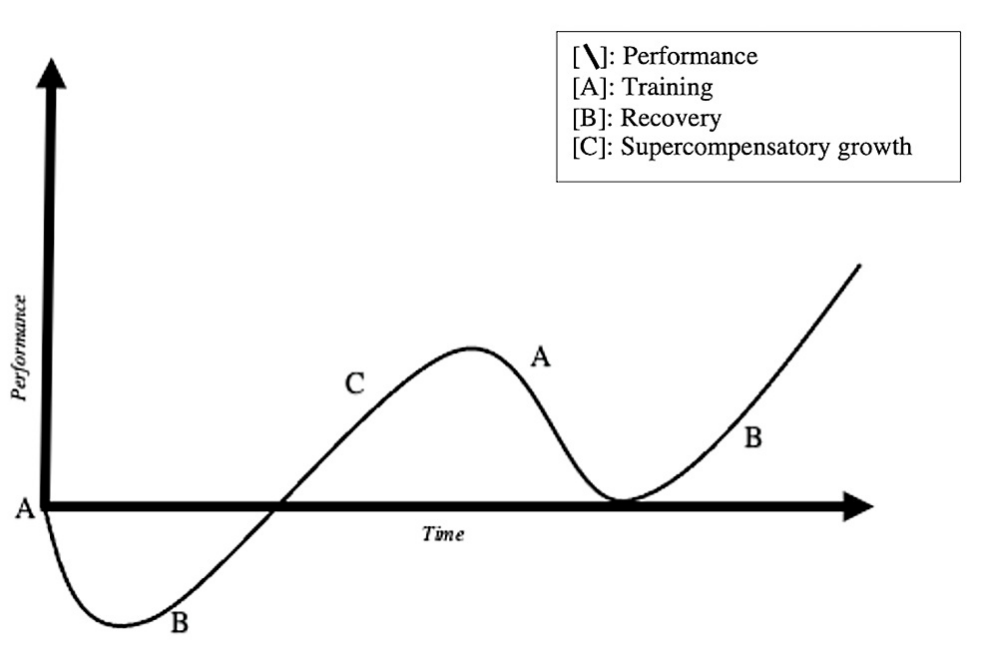
Performance curve. Performance is depicted as the y-axis, with time as the x-axis. Training session (A) leads to fatigue and decreased performance. Given resources and rest, recovery (B) ensues. Following a sufficient stimulus and adequate recovery period, a supercompensatory increase (C) in performance develops. The process repeats with a subsequent training session (A) and recovery (B).

As illustrated below, the act of training, whether physical or mental, temporarily decreases performance. It is the rest and recovery after training that leads to the compensatory increase in performance. In sports, it is known that without adequate resources and recovery, the path to overtraining syndrome (OTS) is likely to begin [11]. Figure 1 shows the sequence of events following a training session (A) given adequate time and recovery (B) opportunity.

Dr. Kreher and Dr. Schwartz describe OTS as a group of symptoms including fatigue, anhedonia, loss of motivation, insomnia, irritability, agitation, lack of mental concentration, tachycardia, unrefreshing sleep, among others [12]. This sounds eerily similar to the non-athletic equivalency, burnout, which includes symptoms such as lack of motivation, exhaustion, cynicism, frustration, inability to cope, depersonalization, and a deterioration of well-being [13]. Due to the zealous focus on the frequency and quantity of training opportunities, the trainee resides on the downward slope, hoping and praying for the opportunity to ascend.

In cognitive training, there is a well-documented decrease in performance corresponding with time-on-task. As mental workload increases, cognitive demand and fatigue increase, resulting in more errors, worsened memory, distraction, attention lapses, and slower reactions [14-17]. These performance decrements associated with a given task load have even been replicated under functional magnetic resonance imaging with alterations in cerebral blood flow [18]. Just like in strength training, the initial training stimulus results in a decrease in performance. The performance trajectory continues to mimic the sports performance model when a trainee is provided with adequate rest and recovery opportunity. Learning opportunity followed by sleep significantly improves retention and recall when compared to an equivalent time awake (e.g., 12 hours awake vs. 12 hours asleep) [19,20]. And, just as strength is not developed in a single, volume-intensive workout, memory is also best broken down into smaller, but repeating sessions. Since the late 1800s spaced repetition has been a consistent beacon of optimal learning practices. When the volume of learning repetitions are controlled, long-term memory retention is consistently greater in spaced repetition groups [21]. The laborious and volume-driven methods of learning are ineffective [22], and when additional time studying is replaced with naps or sleep, performance improves [23].

**Figure 2:**
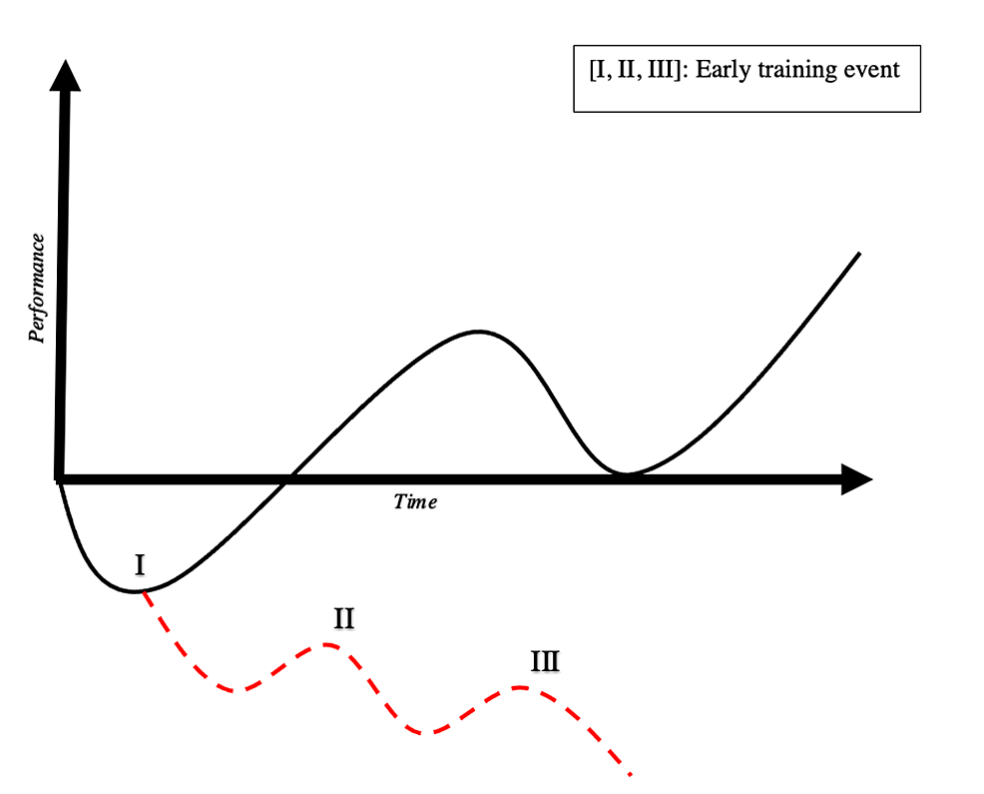
Performance curve with early training. Adapted from Figure 1, time (x-axis) and performance (y-axis) are again depicted graphically. Performance (solid line) initially decreases, then with rest and recovery, a super-compensatory increase in performance above baseline. Event [I], [II] and [III] represent subsequent training prior to recovery. An additional shift, on-call duty, resiliency workshop, or other similar events limit the beneficial effects of the initial training and maintain a sub-baseline performance outcome.

Shifting the framework to encourage the incorporation of ergogenic principles into fields beyond athletics can be accomplished by understanding the concept of overtraining and its impact on development. Figure 2 illustrates the outcome of various events following training and its impact on subsequent performance. Prior to the compensatory improvement as a result of the training, an additional training session, sleep deprivation, lack of nutritional or other resources can cause the “performance line” to continue its downward trajectory.

## Results

A petri dish for growth

Hardworking, intelligent, motivated, and driven are oft-cited attributes of medical students. Yet, the focus of current interventions involves targeting these inherent traits. If the right culture and environment are created, a physician burgeoning can occur. In this light, the question of medical residency and education transitions from “what needs to be added?” to “what is hindering growth?” Of noteworthy importance, the answer is not centered around the removal of hardship and stress, it is about the subtraction of obstacles limiting the rest and recovery time that is necessary to enjoy the fruits of labor.

Without making too strong of assumptions, the pursuit of doctoral-level education in itself signifies hard work and dedication. Sleep deprivation, the liminal rite-of-passage mentality, and an absolute dearth of free time takes an “elite” learner and maims them. Imagine if this population of innately driven students is placed in an environment that stokes growth and centers around performance instead of a focus on only one half of the equation, work. This is not moral outrage regarding “overworked” physicians. The path to becoming a leader in medicine involves stress, work, and uphill battles, but it must be understood that this is the period performance is decreasing. It is not considered growth until the integration and compensatory stage that follows when provided the right environment. There is a sense of injustice when the opportunity to recover and ascend is not provided after training. To tie back in the hypertrophy analogy, what good is the workout without the gains?

The pendulum swing

Researchers Rudland, Golding, & Wilkinson discuss a paradoxical and semantic relationship with “stress” [24]. While the current paper expresses shared sentiment regarding the definition and need of stress, there is a fundamental difference that must be addressed. First, it is with great caution that we promote stress as providing a beneficial role in education. It is likely the concept of eustress and the need for a stressor to grow that has led to commonly held societal myths such as “what doesn’t kill me, makes me stronger.” As briefly discussed regarding muscle hypertrophy, stress is necessary, but any conversation built around stress must include the compensatory period after stress in which any beneficial effects occur.

Second, as many in the medical field can attest to, duration is a key concept in determining benefits or harm. Acute stress with adequate recovery is necessary to produce beneficial and super-compensatory adaptations. Chronic stress or stress without recovery, on the other hand, is the “bad stress” Rudland et al. mention [24]. One must look no further than inflammation to illustrate the concept. In acute bouts of inflammation following an injury or infection, the inflammatory cascade is immensely beneficial and needed. Inflammation without resolution, however, leads to degeneration and improper healing. The temporal scale of stress is often missing when discussing its utility and role.

Third, the lack of consensus regarding the role of stress and learning outcomes can be explained physiologically. Susanne Vogel & Lars Schwabe (2016) interpret stress and learning research with a review on the underpinning mechanisms involved in stress. In short, the two major stress pathways in the body consist of an immediate reaction (noradrenaline) and a slower acting but longer duration reaction (cortisol) [25]. The timing and duration of the stress influence memory retention, cognition, and exam performance [25].

Entrapping stress as a positive and necessary attribute of medical education takes the learning environment on a pendulum swing that only pauses at the poles. 80-hour work weeks to optimize exposure opportunities is evidence of the binary solution that more work is better. Progress and growth demand work and stress, this is the critical signaling to which we can compensate and adapt in a given environment. If work and recovery exist on the respective ends of a spectrum, the current balance is markedly skewed towards work. Introducing nuance to the development of future physicians can create a more balanced ratio of work:recovery. Therefore, the insistence of increasing training hours and exposure opportunities can only be used in a positive light if adequate recovery opportunity is accompanying.

## Discussion

The contradiction of training visions

Briefly, the Accreditation Council of Graduate Medical Education (ACGME) is the agency that develops the standards to develop physicians into the leaders in medicine each aspires to be [26]. Among others, ACGME visions statements include building an environment with competency-based professional development, clinical immersion in “care environments defined by excellence in clinical care…” and promoting graduates who continue to strive for mastery and altruism [26]. As stated earlier, there is a plethora of literature on burnout rates and mental health declines in medical residency. There is a broken methodology if the outcome is so far distanced from the mission. The tantamount definitions and pathophysiology of burnout and overtraining syndrome indicate the applicability to translate athletic ergogenic techniques into education curricula to better meet the ACGME visions.

The hyper-focus on competency-based metrics dominates an aspiring physician’s education career. Yet, the all-too-common sleep deprivation as a result of training demand decelerates progression. Prior to each shift, the brain needs a “pre-treatment” of sleep to incorporate the upcoming lessons and clinical pearls. Next day learning is impaired with just one night of sleep deprivation leading to a hindered ability to incorporate the lessons of today [27,28]. In fact, not only is the hippocampal impairment well-studied as a victim of sleep loss [29], but sleep deprivation can also lead to false memories [30]. More, shift-work induced circadian misalignment significantly impacts attention, learning, and information processing, in which sustained attention does not recover until three days following a shift back to a circadian aligned schedule [31]. With a competency-based vision, the current environment escorts physicians in an opposing direction.

Likewise, as discussed earlier, the symptoms of overtraining syndrome and the equivalent burnout, lead to loss of motivation [32,33]. This is in direct contrast to mission statements that emphasize motivational aspects of behavior such as the continual strive for mastery. A meta-analysis from 2018 by Rodrigues et al. reports a burnout prevalence of 35.7% among all specialties [32]. The paradoxical relationship of the outcome and vision should serve as an indication of a necessary overhaul in the curriculum. One feature that is aligned with the vision of graduate medical education standards is the attempt to prioritize physician well-being. Unfortunately, curriculum additions that target mindfulness and resiliency training [33-35] only serve to perpetuate the myth that more training is the missing link to improve health and well-being.

## Conclusions

The correction

The “too long; didn’t read” synopsis can be summed up with three words: training decreases performance. It does, however, serve as the necessary signal to facilitate growth and improvement. In order for this signal to flourish, there is a need for adequate recovery time and resources. Medical training, like many educational curriculums, focus on one half of the spectrum (work) which prioritizes exposure, hours, and other quantity-based metrics. The work:recovery ratio is heavily weighted to the work side, which without adequate recovery, negates the preceding effort, exposure, and duty hours.

Using the public health ethic of health maximization as a foundation for guiding curriculum changes, the pool of innate talent can be amplified. One way to create this modification is to ask what is hindering health maximization? In the fractional sub-population of the United States in pursuit of doctoral-level education, the answer is not a lack of resiliency, dedication, or mindfulness. When these traits are assumed as a given, the anchor that is slowing growth and creating burnout or burnout symptoms can be logically assumed to be environmentally caused. By viewing medical training, or any training for that matter, from the sports performance perspective, ergogenic techniques can be applied that emphasize rest in chronically under-recovered populations. An intense, long, or difficult shift can only lead to the intended benefits if it is followed by an adequate recovery period. The super-compensatory hop after training or learning opportunities can be used to augment the resources and attributes currently in place in a graduate medical education curriculum.
